# Prevalence and correlates of hazardous alcohol consumption and binge drinking among men who have sex with men (MSM) in San Francisco

**DOI:** 10.1371/journal.pone.0202170

**Published:** 2018-08-17

**Authors:** Glenn-Milo Santos, Christopher Rowe, Jaclyn Hern, John E. Walker, Arsheen Ali, Marcial Ornelaz, Maximo Prescott, Phillip Coffin, Willi McFarland, H. Fisher Raymond

**Affiliations:** 1 Center for Public Health Research, San Francisco Department of Public Health, San Francisco, California, United States of America; 2 Department of Community Health Systems, School of Nursing, University of California San Francisco, San Francisco, California, United States of America; 3 School of Public Health, University of California Berkeley, Berkeley, California, United States of America; 4 The Henry M. Jackson Foundation for the Advancement of Military Medicine, Bethesda, Maryland, United States of America; 5 Division of HIV, ID & Global Medicine, University of California, San Francisco, California, United States of America; 6 Department of Epidemiology and Biostatistics, School of Medicine, University of California San Francisco, San Francisco, California, United States of America; 7 Department of Epidemiology, Rutgers School of Public Health, Piscataway, New Jersey, United States of America; University of Rochester School of Medicine and Dentistry, UNITED STATES

## Abstract

**Objectives:**

To describe heavy alcohol use patterns and correlates in a diverse sample of MSM.

**Methods:**

We used respondent-driven sampling (RDS) to enroll 252 alcohol-using MSM in San Francisco from March 2015-July 2017. We examined heavy alcohol use patterns and conducted RDS-adjusted multivariable analyses to characterize correlates of hazardous alcohol consumption and binge drinking.

**Results:**

RDS-adjusted prevalence of weekly and at least weekly binge drinking was 24.9% and 19.3%, respectively. Hazardous consumption was common; prevalence of mid- and high-levels of hazardous drinking was 11.4% and 29.9%, respectively. In multivariable analyses, identifying as Hispanic/Latino or mixed/other race; being moderately or extremely interested in reducing alcohol use; ever receiving alcohol treatment; using ecstasy; reporting syphilis diagnosis; and having more than 5 male partners were independently associated with hazardous alcohol consumption. Less hazardous consumption was associated with having a bachelor's degree or completing post-graduate studies; and not being in a relationship. Reporting chlamydia infection; being somewhat, moderately or extremely interested in reducing alcohol use; and having multiple male sex partners were associated with higher odds of at least weekly binge drinking. Lower odds of binge drinking were associated with completing post-graduate studies. Moreover, for the outcomes of hazardous alcohol consumption and binge-drinking, we observed significant interaction effects between race/ethnicity and interest in reducing alcohol, past receipt of alcohol treatment, use of ecstasy, syphilis diagnosis, and number of male partners.

**Conclusion:**

Among alcohol-using MSM in San Francisco, heavy drinking patterns were common and independently associated with greater number of male sexual partners and sexually transmitted infections (STI). Moreover, significant racial/ethnic and socioeconomic disparities related to heavy alcohol use were observed and race/ethnicity modified the effect of the risk factors associated with these outcomes. These findings underscore the need to develop more MSM-specific interventions that jointly address heavy alcohol use and HIV/STI risk, as well as culturally-tailored and targeted strategies to alleviate health disparities.

## Introduction

Heavy alcohol use, including binge drinking (i.e., five or more drinks in a single occasion for men) and hazardous alcohol consumption (i.e., as classified by the alcohol use disorders identification test [AUDIT]), has been linked to a wide range of negative health problems [1,2] and is the fourth leading lifestyle-related cause of death in the United States (US) [3]. Nevertheless, identification and screening for heavy alcohol use remains insufficient; the Centers for Disease Control and Prevention estimate that only one in three US adults are asked about binge drinking, and one in three binge drinkers are screened and advised about harmful drinking by their health providers [4]. Furthermore, uptake of evidence-based alcohol interventions are persistently low: less than one-fourth of individuals with alcohol use disorders receive treatment and fewer than ten percent receive pharmacotherapy [5,6]. Therefore, evaluating the prevalence and correlates of heavy alcohol use is of high public health importance, particularly for high-risk populations.

Studies have described the prevalence and examined the demographic, social and clinical correlates of alcohol use disorders for the general adult population using large epidemiologic surveys. These analyses were guided by the exploratory data analysis conceptual framework with the goal of identifying potential health disparities among racial/ethnic and age groups, identify comorbidities related to heavy alcohol use, and ultimately inform the planning and delivery of public health services [6,7]. For men who have sex with men (MSM), multiple epidemiologic studies have shown a high prevalence of heavy alcohol use [8,9]. Furthermore, heavy alcohol consumption patterns are independently associated with condomless anal intercourse [10,11] and HIV infection among MSM [12–14]. Nevertheless, despite the high prevalence of heavy alcohol consumption and its linkages with HIV infection, few studies have recruited exclusive samples of alcohol-using MSM outside of treatment settings [15]. Most studies involve comparisons between drinkers versus non-drinkers or sub-group analyses of alcohol users, and typically capture limited information on alcohol use patterns [16–18]. Furthermore, we are unaware of MSM-specific studies that have explored the demographic, social, and clinical correlates of heavy alcohol use patterns, similar to those conducted among the adult general population. More empirical studies are needed to further explore the wide range of drinking patterns among alcohol-using MSM, and inform planning and delivery of services for these individuals.

In addition, the majority of studies evaluating alcohol use among MSM to date have used convenience or venue-based samples that may oversample participants from alcohol-serving venues [15,16], which tend to have heavier alcohol users. For example, National HIV Behavioral Surveillance (NHBS) data among MSM in San Francisco observed that men recruited in venues that serve alcohol report greater frequency of binge drinking, number of drinking days, and number of drinks in a typical drinking day [8]. Another concern with venue-based recruitment is the decline in attendance at “physical gay spaces”, such as clubs and bars, as increasing number of MSM turn to “virtual gay communities” (e.g., social-networking spaces within internet and mobile applications)[19–21]. Additionally, recruiting diverse MSM samples in studies has proved challenging for researchers, who have reported difficulties in enrolling youth and men of color, limiting external validity [22]. Taken together, these issues highlight the increasing need to employ a variety of recruitment approaches to reach diverse groups of alcohol-using MSM.

Respondent-driven sampling (RDS) can generate more diverse study populations and has recruited MSM and substance-using populations, whom may be hard to reach and for whom sampling frames do not exists [23–27]. RDS uses peer-recruitment chains to tap into the social networks of study participants [27]. Specifically, it leverages social connections between members of a target study population to communicate study goals and recruit peers into the study. Hence, RDS may mitigate recruitment challenges by potentially reaching members of MSM networks who may not attend traditional physical venues where recruitment has historically occurred [8,28]. Although RDS methods have been used to recruit specific sub-groups, including among alcohol- and drug-using youth [29], ecstasy users [30], illicit stimulant drug users in rural settings [31], and different MSM subpopulations (e.g., MSM international travelers, black MSM, and young MSM [24–26,32]), we are not aware of any RDS studies conducted exclusively among alcohol-using MSM.

The objectives of this study were: 1) to evaluate the prevalence of heavy alcohol use patterns, and 2) explore correlates of heavy alcohol consumption, specifically hazardous drinking and binge drinking, in a diverse sample of alcohol-using MSM recruited by RDS. The second objective involved exploratory analyses to assess which demographic (e.g., age, race/ethnicity), social (e.g., education, income), behavioral (e.g., sexual risk behaviors) and clinical characteristics (e.g., sexually transmitted diseases, depressive symptoms) are associated with heavy alcohol use. Consistent with prior studies completed among the general adult population [6,7], the second objective was guided by the need to identifying sub-populations who may have higher burden of harmful drinking patterns and therefore would be ideal for more targeted screening and referrals to evidence-based interventions. Additionally, we sought to also explore whether certain negative health conditions and behaviors are associated with harmful drinking, which may inform the development of programs to address the needs for heavy drinkers.

## Methods

### RDS recruitment

We recruited participants in a cross-sectional study, entitled *The SEEDS Study*, for alcohol-using MSM using RDS. Initial RDS study “seeds”—the participants who initiate peer-recruitment for their networks—were selected to reflect a diverse sample of MSM across age, race/ethnicity, education, and income (n = 11). As recruitment slowed, additional seeds were identified and added (n = 13) to sustain enrollment, which is consistent with RDS methodology and other MSM studies [25–27]. Seeds were screened during an in-person interview and were invited to participate based on their willingness to recruit and motivate their peers to participate in the RDS study. Eligible seeds were given recruitment coupons and were asked to recruit as many as 5 participants, who in turn were asked to recruit a subsequent wave of participants, and so on, until sample size was reached and equilibrium was achieved on key variables. Recruitment coupons included unique tracking numbers that allowed the study to link which seed/participants referred new participants. All seeds and participants who utilized all their coupons were offered additional coupons (no more than 8 total, including the initial coupons distributed; this higher coupon limit is consistent with another MSM study [25]). As recruitment progressed, coupon distribution was adjusted based on accrual of demographic subgroups in the study (i.e., race/ethnicity and neighborhood) and the stage of the study recruitment (i.e., final wave of study participants received 0 coupons).

### Study procedures

Study participants (including seeds) were screened for eligibility before enrollment. Individuals were eligible if they reported (1) their sex assigned at birth as male or current gender identity as male, (2) having sex with at least 1 man in the past 12 months, (3) being aged 18 years or older, (4) alcohol use in the past 12 months, and (4) living in the San Francisco Bay Area. If eligible, staff obtained signed consent using an institutional research board (IRB)-approved form. After consenting, participants completed behavioral surveys lasting approximately 30 minutes using audio computer assisted self-interview (ACASI). After completing the ACASI, study staff provided participants with referral coupons and provided participants with basic peer-recruitment techniques. Participants received $30 for their enrollment visit and received an additional $10 for each person they referred into the study who completed the enrollment visit. Participants who successfully enrolled their peers into the RDS study were also entered into monthly raffles, with prizes ranging from $50–100 gift cards, consistent with incentives strategies used in another MSM RDS study in San Francisco [25]. All study procedures and study materials were reviewed and approved by the University of California San Francisco IRB (IRB study #14–14481).

### Behavioral questionnaire

ACASI was used to standardize data collection and minimize reporting bias for a range of demographic, social, and behavioral measures, including alcohol use and sexual risk behaviors [33–35]. Hazardous alcohol consumption was evaluated using the World Health Organizations’ Alcohol Use Disorders Identification Test (AUDIT), a 10-item measure used to screen for hazardous alcohol use. AUDIT scores were calculated and dichotomized based on a cut-off of 16; individuals with a score of 16 or greater are considered to have a mid- to high-level risk of problem drinking (“hazardous”)[36]. Participants were also asked to respond to a range questions related to their alcohol use patterns, goals they may have pertaining to their alcohol use (if any), and alcohol treatment history. Participants who reported at least weekly binge drinking and expressed interest in cutting down their alcohol use were subsequently invited to screen for another ongoing pharmacotherapy study to reduce heavy episodic alcohol use conducted at the San Francisco Department of Public Health [37]. Data in S1 File.

### Data analyses

We conducted analyses using STATA version 14 (College Station, TX) and a statistical program for analyzing RDS study data, RDS Analyst (RDS-A) version 0.42 [38]. In RDS-A, we generated individualized sampling weights using the RDS-II estimator. The RDS-II estimators use a Markov chain model to create weight estimates for the probability of inclusion of each individual into the study, based on their reported social network size [39]. Social network size was imputed as the median (median = 20) for individuals with missing network size (n = 19). RDS-adjusted prevalence and 95% CI of the participant characteristics were estimated in RDS-A bootstrapped models, except when prevalence estimates only applied for sub-group of study participants (e.g., types of alcohol treatment received among those who reporting receiving treatment). For the latter case, RDS-adjusted prevalence were estimated in STATA using exported sampling weights from RDS-A.

For bivariate and multivariable analyses, individualized RDS-II weights were exported from RDS-A and merged with the ACASI dataset in STATA. We then used the weights in STATA to conduct RDS-adjusted bivariate and multivariable logistic regression analyses to explore the demographic, behavioral, and clinical correlates for the following heavy alcohol use outcomes of interest: (1) hazardous alcohol consumption and (2) at least weekly binge drinking. For model building, characteristics that had a p-value <0.25 in bivariate models were considered for the full multivariable models, which were then finalized using a stepwise, backward elimination approach. The final model retained correlates with overall p-values<0.25 as assessed using Wald Tests, as well as key potential demographic and clinical confounders (race/ethnicity, age, education, and having clinically significant depressive symptoms). Race/ethnicity, age, education, and depressive symptoms were selected for inclusion *a priori* because there are established associations between these characteristics and alcohol use [40,41].

Because there are documented differences in alcohol use patterns as well as disparities in health and social consequences of alcohol use by race/ethnicity [41,42], we also assessed interaction effects between race/ethnicity and key participant characteristics. Specifically, we included interaction terms between race/ethnicity and each characteristic that was significantly associated with the outcome in the main multivariable models. Interaction effects between race/ethnicity and each significant characteristic were assessed in separate models due to data sparsity that would result from the inclusion of multiple sets of interaction terms in a single model. Models included all covariates that were included in the main multivariable model for each respective outcome. The overall significance of the interaction effects between race/ethnicity and each individual participant characteristic for a given outcome was assessed using Wald Tests; for overall interaction effects that were significant at p < 0.25, we calculated race/ethnicity specific effects for the appropriate participant characteristic. Interaction effects were assessed for both hazardous alcohol consumption and weekly or more binge drinking. Due to a lack of outcome variability among specific race/ethnicity-covariate combinations, “not” and “somewhat” interested in reducing amount of alcohol consumed were collapsed and used as the reference level in the model assessing interaction effects on hazardous alcohol consumption and zero and one male sex partners were collapsed and used as the reference level in the model assessing interaction effects on weekly or more binge drinking. There were no significant differences between the collapsed categories in the relevant multivariable model.

## Results

### Seed and recruitment chain characteristics

Including the 24 seed participants, the final crude sample consisted of 252 alcohol-using MSM. We provide detailed characteristics of seeds in Table 1. The majority of seeds had a bachelor’s degree (n = 13) and were between the ages of 18–34 (n = 19) years.

**Table 1:** Seed characteristics, alcohol-using men who have sex with men: San Francisco, CA; March 2015—June 2017 (n = 24).

Seed ID	Race/Ethnicity	Age, Years	Education	Income, $	Recruits, No.	Waves, No.
A	Hispanic/Latino	18–34	Bachelor's Degree	$40,000–$74,999	9	2
B	Black/African American	18–34	Some College or 2 Year Degree	$0–$9,999	2	1
C	White	34–50	Bachelor's Degree	$75,000+	1	1
D	Hispanic/Latino	18–34	Some College or 2 Year Degree	$75,000+	0	0
E	Asian/Pacific Islander	34–50	Bachelor's Degree	$75,000+	13	4
F	White	18–34	Any Post-Graduate Studies	$75,000+	3	2
G	White	34–50	Bachelor's Degree	$75,000+	1	1
H	Mixed/Other	18–34	Some College or 2 Year Degree	$20,000–$39,000	122	19
I	Asian/Pacific Islander	18–34	Any Post-Graduate Studies	$75,000+	0	0
J	White	18–34	Bachelor's Degree	$40,000–$74,999	2	1
K	Asian/Pacific Islander	18–34	Bachelor's Degree		0	0
L	Hispanic/Latino	18–34	Bachelor's Degree	$75,000+	1	1
M	Black/African American	18–34	Bachelor's Degree	$40,000–$74,999	0	0
N	White	18–34	Bachelor's Degree	$75,000+	0	0
O	Hispanic/Latino	18–34	High School or GED	$20,000–$39,000	7	2
P	White	18–34	Any Post-Graduate Studies	$40,000–$74,999	0	0
Q	White	18–34	Bachelor's Degree	$20,000–$39,000	0	0
R	Hispanic/Latino	34–50	Some College or 2 Year Degree	$75,000+	5	2
S	Asian/Pacific Islander	18–34	Bachelor's Degree	$75,000+	31	7
T	Hispanic/Latino	18–34	Some College or 2 Year Degree	$40,000–$74,999	0	0
U	White	18–34	Bachelor's Degree	$40,000–$74,999	0	0
V	Mixed/Other	51+	Some College or 2 Year Degree	$40,000–$74,999	28	8
W	Hispanic/Latino	18–34	Bachelor's Degree	$75,000+	0	0
X	White	18–34	Any Post-Graduate Studies	$10,000–$19,999	3	1

**Fig 1:**
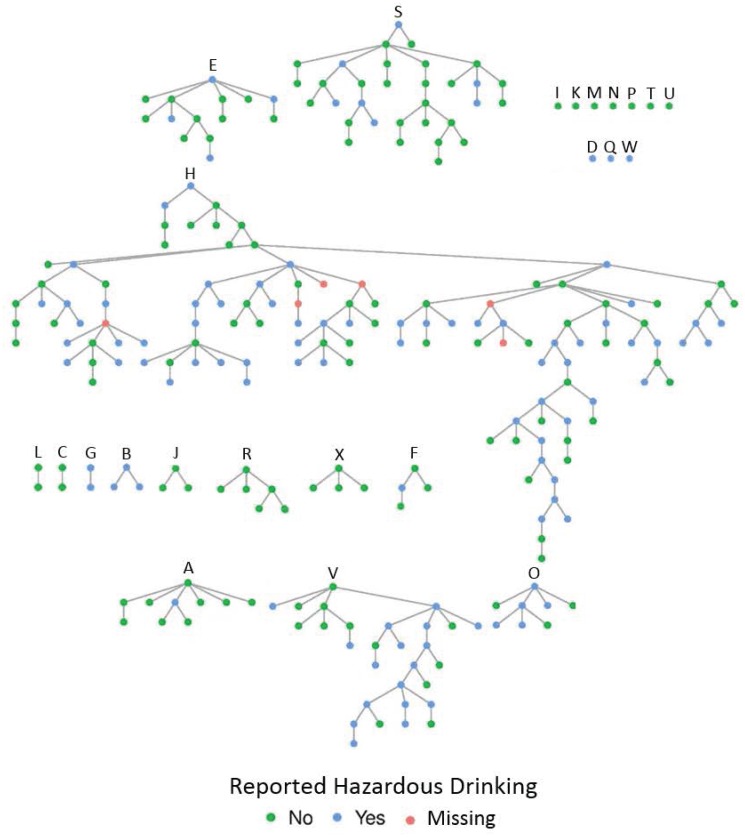
Participant recruitment chain of alcohol-using men who have sex with men in San Francisco in The SEEDS Study (n = 252). Letters (A-X) correspond with 24 initial participants (“seeds”) selected by study staff to initiate peer-recruitment (see Table 1 for characteristics of seeds). Hazardous drinking defined using a score cut-off of 16 in the alcohol use disorders identification test (AUDIT).

As in many RDS studies, the majority of recruitment stemmed from a few seeds [25,43]. As shown in the social network diagram in Fig 1, seed H produced the majority of recruits (n = 122), seed S produced 31 recruits, and seed V produced 28 recruits. These 3 chains were reasonably long at 19, 7, and 8 waves, respectively. The mean network size reported by race/ethnicity is shown in Table 2. Excluding missing values that were imputed to the median, mean network size by race/ethnicity ranged from 18.3 to 81.7, for African Americans and Mixed/Other race, respectively.

**Table 2:** Seed, sample, and estimated population proportions, and mean network size, by race/ethnicity, alcohol-using men who have sex with men: San Francisco, CA; March 2015—June 2017.

Race/Ethnicity	Seeds, %	Sample, %	Estimated Population, %	Network Size, Mean
Asian/Pacific Islander	16.7%	13.1%	7.5%	68.0
Black/African American	8.3%	31.0%	46.6%	18.3
White	37.5%	32.9%	31.2%	44.7
Hispanic/Latino	29.2%	15.1%	10.8%	71.3
Mixed/Other	8.3%	7.9%	4.0%	81.7

Recruitment of the entire sample occurred between March 2015 and July 2017 with a mean of 3.7 recruitment waves for active seeds (range = 1–19). The overall coupon return rate was 33.6%; excluding unproductive seeds, the coupon return rate was 35.7%. A total of 268 individuals were presented with a recruitment coupon (i.e., non-seeds) and screened; of these, 228 (85.1%) were eligible and enrolled, 22 (8.2%) were eligible and lost to follow-up, one (0.4%) was eligible and declined to participate, and 17 (6.3%) were ineligible. We examined recruitment patterns for key variables (e.g., race/ethnicity, age, education) and found that in general the sample converged on the final sample composition and we found no evidence of bottlenecks.

### Participant characteristics

Study participants were diverse with respect to race/ethnicity, age, and sociodemographic characteristics, as shown in Table 3. Most participants were MSM of color (RDS-adjusted prevalence: 46.6% African American, 10.8% Hispanic/Latino; 7.5% Asian and Pacific Islander), had some college (37.2%), and were ages 18–50 (age 18–34 = 28.4%; 35–50 = 36.2%). Most participants were HIV-negative (61.5%), only had sex partners who were male (65.1%), had condomless anal intercourse in the past 6 months (58.1%), and were single/not in a relationship (66.4%).

**Table 3:** Crude and RDS-weighted characteristics among alcohol using men who have sex with men: San Francisco, CA; March 2015—June 2017 (n = 252).

	Crude	Weighted
Characteristic	n (%)	% (95% CI)
Total	252	
**DEMOGRAPHIC CHARACTERISTICS**		
**Race/Ethnicity**		
Asian/Pacific Islander	33 (13.1)	7.5 (0.0, 20.3)
Black/African American	78 (31.0)	46.6 (40.4, 52.8)
White	83 (32.9)	31.2 (23.0, 39.4)
Hispanic/Latino	38 (15.1)	10.8 (0.0, 21.9)
Mixed/Other	20 (7.9)	4.0 (0.0, 12.9)
**Age, y**		
18–34 (Millenials)	102 (40.5)	28.4 (16.9, 40.0)
35–50 (Generation X)	75 (29.8)	36.2 (24.6, 47.9)
51+ (Baby Boomers)	75 (29.8)	35.3 (23.5, 47.2)
**Education**		
< High school	20 (7.9)	14.1 (10.5, 17.7)
High School or GED	53 (21.0)	26.2 (11.0, 41.4)
Some College or 2 Year Degree	83 (32.9)	37.2 (36.4, 38.1)
Bachelor's Degree	61 (24.2)	14.3 (8.0, 20.6)
Any Post-Graduate Studies	33 (13.1)	5.9 (0.0, 15.3)
**Employment status**		
Not employed	124 (49.2)	65.3 (62.3, 68.3)
Employed	115 (45.6)	28.9 (14.9, 43.0)
Student, with or without part-time employment	10 (4.0)	3.4 (0.2, 6.6)
**Field of Employment**		
Healthcare and social assistance	25 (9.9)	5.5 (2.7, 8.3)
Technology	21 (8.3)	3.9 (2.7, 5.1)
Accomodation and Food Services	10 (4.0)	2.7 (0.0, 10.6)
Educational Services	7 (2.8)	1.3 (0.0, 11.2)
Finance and Insurance	6 (2.4)	0.6 (0.0, 2.1)
Retail	6 (2.4)	0.6 (0.0, 5.3)
Other	47 (18.7)	17.1 (13.0, 21.3)
**Income (yearly), $**		
< 10,000	82 (32.5)	44.0 (31.8, 56.2)
10,000–19,999	39 (15.5)	18.7 (10.3, 27.0)
20,000–39,999	42 (16.7)	16.0 (6.9, 25.1)
40,000–74,999	28 (11.1)	10.4 (3.2, 17.5)
≥ 75,000	51 (20.2)	7.8 (1.8, 13.8)
**Born in the United States**	231 (91.7)	91.4 (91.2, 91.6)
**Currently homeless**	69 (27.4)	34.1 (34.1, 34.1)
**Relationship status**		
In a relationship	85 (33.7)	31.2 (19.1, 43.2)
Single	158 (62.7)	66.4 (62.2, 70.6)
Other	8 (3.2)	2.2 (0.0, 4.7)
**Alcohol-using MSM network size (quartiles)**		
Quartile 1: 1–8	60 (23.8)	66.7 (56.5, 76.9)
Quartile 2: 9–20	67 (26.6)	20.8 (14.0, 27.7)
Quartile 3: 21–50	52 (20.6)	6.3 (0.0, 13.5)
Quartile 4: 51–800	54 (21.4)	2.2 (0.0, 9.8)
**ALCOHOL USE**		
**Frequency of alcohol consumption, past 12 mo**		
Monthly or less	14 (5.6)	15.7 (11.0, 20.4)
2–4 times per month	44 (17.5)	21.7 (9.1, 34.3)
2–3 times per week	94 (37.3)	23.0 (9.4, 36.5)
4 or more times per week	100 (39.7)	39.7 (27.6, 51.7)
**Frequency of binge alcohol consumption, past 12 mo**		
Never	28 (11.1)	12.9 (6.7, 19.1)
Less than monthly	59 (23.4)	29.0 (20.4, 37.7)
Monthly	46 (18.3)	13.7 (7.2, 20.2)
Weekly	78 (31.0)	24.9 (24.5, 25.2)
Daily or almost daily	40 (15.9)	19.3 (16.1, 22.5)
**AUDIT Score (Risk Level)**		
0–7 (Zone I)	46 (18.3)	20.9 (12.5, 29.4)
8–15 (Zone II)	96 (38.1)	36.3 (26.6, 45.9)
16–19 (Zone III)	36 (14.3)	11.4 (4.1, 18.7)
20–40 (Zone IV)	68 (27.0)	29.9 (26.6, 33.2)
**Severity of Dependence Score of 3 or above**	116 (46.0)	51.7 (51.7, 51.7)
**Used alcohol delivery mobile application, past 12 mo**	25 (9.9)	15.8 (15.7, 15.9)
**Alcohol use during any sex, past 4 wk**		
Never	62 (24.6)	24.5 (19.4, 29.6)
1–25% of the time	95 (37.7)	40.4 (34.6, 46.2)
26–50% of the time	34 (13.5)	12.9 (5.7, 20.2)
51–75% of the time	16 (6.3)	4.1 (0.0, 12.2)
76–100% of the time	28 (11.1)	13.0 (4.1, 21.8)
No recent anal sex	16 (6.3)	4.9 (4.9, 4.9)
**Current alcohol-related goal**		
No clear goal	77 (30.6)	28.8 (23.9, 33.7)
Use alcohol in controlled manner	102 (40.5)	44.1 (39.3, 48.9)
Temporary abstinence	15 (6.0)	7.7 (5.9, 9.6)
Occasional but not habitual alcohol use	13 (5.2)	3.8 (0.0, 15.4)
Permanent abstinence, with expected possibility of relapse	20 (7.9)	6.0 (4.0, 8.1)
Permanent abstinence, with no expected possibility of relapse	10 (4.0)	4.5 (0.4, 8.5)
Other	12 (4.8)	4.4 (0.0, 10.3)
**Interest in reducing amount of alcohol consumed**		
Not interested	71 (28.2)	31.7 (26.8, 36.7)
Somewhat interested	91 (36.1)	38.3 (37.1, 39.5)
Moderately interested	47 (18.7)	15.8 (6.2, 25.4)
Extremely interested	42 (16.7)	14.0 (6.3, 21.7)
**Ever had an alcohol-related hospitalization**	52 (20.6)	21.1 (21.1, 21.1)
**Ever tried to stop or reduce alcohol use**	179 (71.0)	65.1 (64.7, 65.4)
**Ever received treatment for alcohol use**	83 (32.9)	36.3 (35.8, 36.8)
**Type of treatment for alcohol use reported**		
Behavioral Treatments	34 (13.5)	14.0 (14.0, 14.0)
Medication/pharmacotherapy	15 (6.0)	3.5 (2.8, 4.1)
Mutual-support groups	57 (22.6)	22.8 (22.5, 23.0)
Other	14 (5.6)	4.2 (2.9, 5.5)
**Type of medication/pharmacotherapy for alcohol use reported**		
Oral naltrexone	5 (2.0)	1.1 (1.1, 1.1)
Injectable naltrexone	3 (1.2)	0.7 (0.6, 0.8)
Disulfiram	6 (2.4)	1.5 (1.4, 1.6)
**OTHER SUBSTANCE USE**		
**Injected drugs, past 6 mo**	41 (16.3)	24.8 (20.6, 29.0)
**Use of specific substances, past 6 mo**		
Methamphetamine	83 (32.9)	38.7 (36.1, 41.2)
Powdered Cocaine	87 (34.5)	22.5 (22.1, 22.8)
Crack Cocaine	42 (16.7)	19.7 (19.7, 19.7)
Ecstasy	68 (27.0)	17.4 (15.3, 19.5)
GHB	41 (16.3)	11.2 (11.2, 11.2)
Ketamine	31 (12.3)	8.5 (8.5, 8.5)
Poppers	87 (34.5)	28.5 (28.5, 28.5)
Viagra	61 (24.2)	16.8 (13.0, 20.6)
Marijuana	156 (61.9)	54.0 (52.4, 55.5)
Cigarettes	125 (49.6)	48.7 (37.4, 60.1)
E-cigarettes	48 (19.0)	14.6 (3.5, 25.7)
**Ever received treatment for drug use**	94 (37.3)	46.1 (43.7, 48.5)
**MENTAL AND PHYSICAL HEALTH**		
**Clinically Significant Depressive Symptoms**	126 (50.0)	54.1 (53.8, 54.4)
**HIV status, self-report**		
Positive	71 (28.2)	32.6 (32.2, 32.9)
Negative	174 (69.0)	61.5 (52.6, 70.4)
Unknown	6 (2.4)	5.8 (5.6, 5.9)
**Undetectable Viral Load**	24 (9.5)	11.1 (2.4, 19.6)
**Number of HIV tests, past 2 yr**		
Zero	48 (19.0)	22.6 (12.8, 32.3)
One to three	81 (32.1)	34.0 (25.9, 42.1)
Four or more	112 (44.4)	36.3 (31.3, 41.3)
**Currently taking PrEP**	45 (17.9)	16.8 (14.6, 18.9)
**HCV status, self-report**		
Positive	27 (10.7)	10.5 (9.2, 11.8)
Negative	221 (87.7)	86.8 (86.6, 87.0)
**Ever had a sexually transmitted infection, past 6 mo**	58 (23.0)	16.8 (13.2, 20.4)
**Type of sexually transmitted infection, past 6 mo**		
Syphilis	15 (6.0)	6.3 (5.4, 7.2)
Gonnorhea	32 (12.7)	6.4 (2.9, 9.8)
Chlamydia	23 (9.1)	4.6 (0.8, 8.4)
Herpes (HSV)	4 (1.6)	0.8 (0.0, 2.6)
Genital warts (HPV)	9 (3.6)	3.2 (2.7, 3.8)
**SEXUAL BEHAVIORS**		
**Gender of sexual partners**		
Men	201 (79.8)	65.1 (55.3, 75.0)
Both Men and Women	51 (20.2)	34.9 (25.0, 44.7)
**Number of sex partners, past 6 mo**		
Zero	8 (3.2)	4.2 (0.0, 16.8)
One	40 (15.9)	15.9 (10.6, 21.3)
2–5	102 (40.5)	50.2 (49.4, 50.9)
6+	97 (38.5)	26.7 (26.1, 27.3)
**Number of male sex partners, past 6 mo**		
Zero	10 (4.0)	8.6 (0.0, 21.3)
One	45 (17.9)	14.8 (12.1, 17.4)
2–5	103 (40.9)	54.0 (53.2, 54.8)
6+	89 (35.3)	19.6 (18.7, 20.5)
**Any condomless anal intercourse, past 6 mo**	171 (67.9)	58.1 (58.1, 58.1)
**Any insertive condomless anal intercourse, past 6 mo**	156 (61.9)	53.8 (53.8, 53.8)
**Any receptive condomless anal intercourse, past 6 mo**	138 (54.8)	48.4 (48.1, 48.6)
**Any serodiscordant condomless anal intercourse, past 6 mo**	83 (32.9)	30.0 (27.3, 32.7)

### Alcohol and other substance use

In the past year, many participants consumed alcohol at least 4 times per week (39.7%), and most reported binge drinking at least weekly (24.9% weekly, 19.3% more than weekly). Hazardous alcohol consumption was common in the study; the RDS-adjusted prevalence of men with mid- (AUDIT score 16–19) to high-level (AUDIT score 20–40) of hazardous drinking in the sample were 11.4% and 29.9%, respectively.

Many participants reported that their current goal regarding their alcohol use was “to use alcohol in a controlled manner” (44.1%), while a considerable proportion reported that they had “no clear goal” (28.8%). A minority reported permanent abstinence as their goal (6% permanent abstinence with expected possibility of relapse; 4.5% permanent abstinence with no expected possibility of relapse). In addition, the majority of participants expressed interest in wanting to reduce the amount of alcohol they consumed (38.3% were somewhat interested, 15.8% moderately interested, and 14% extremely interested). The majority (65.1%) reported a lifetime history of trying to stop or reduce their alcohol use, although only 36.3% had ever received treatment for alcohol use.

In the overall sample, the most common alcohol treatment program used was mutual-support groups (e.g., Alcoholics Anonymous), followed by behavioral treatments (e.g., psychotherapy), which were reported by 22.8% and 14% of participants, respectively. Few participants reported using medications (pharmacotherapy) for alcohol treatment (3.5%). Among the subset of participants who received alcohol treatment, 62.8% (95% CI: 45.6%-77.2%) reported participating in mutual support groups, 38.6% (24.4%-55.0%) reported receiving behavioral treatment, and 9.5% (4.7%-18.3%) reported utilizing pharmacotherapy. Among the subset of participants with hazardous alcohol consumption, 55.4% (40.8%-69.1%) had received treatment for alcohol use, with 6.9% (3.2%-14.2%) having received pharmacotherapy (data not shown in table).

In the past 6 months, 25% of alcohol-using MSM reported injecting drugs. Moreover, many MSM reported using other non-injection substances recreationally in the past 6 months, including: marijuana (54%), cigarettes (48.7%), methamphetamine (38.7%), poppers (28.5%), cocaine (22.5%); crack (19.7%); ecstasy (17.4%), sildenafil (Viagra) (16.8%), e-cigarettes (14.6%), GHB (11.2%), and ketamine (8.5%).

### Bivariate analyses

As shown in Table 4, hazardous alcohol consumption was correlated (p<0.05 for at least one category) for race/ethnicity, education, income, housing status, interest in reducing the amount of alcohol consumed, substance use (cocaine, crack, ecstasy), receiving substance use treatment, depressive symptoms, reporting sexually transmitted infection (STI) diagnoses (syphilis or any STI), number of sexual partners, and male sexual partners in bivariate analyses. In addition, at least weekly binge drinking was correlated with race/ethnicity, education, interest in reducing the amount of alcohol consumed, HCV status, recent STI diagnoses (syphilis, chlamydia, or any STI diagnosis), number of sexual partners, and male sexual partners in bivariate analyses.

**Table 4:** RDS-Weighted bivariate associations with hazardous alcohol consumption and weekly or more frequent binge drinking among alcohol using men who have sex with men: San Francisco, CA; March 2015—June 2017.

Characteristic	Hazardous Alcohol Consumption			Weekly or More Frequent Binge Drinking		
	OR (95% CI)	*p*-value	Wald *p*-value	OR (95% CI)	*p*-value	Wald *p*-value
**DEMOGRAPHIC CHARACTERISTICS**						
**Race/Ethnicity**						
White	Reference		0.002	Reference		0.018
Black/African American	1.39 (0.51–3.74)	0.517		0.80 (0.31–2.11)	0.657	
Asian/Pacific Islander	0.13 (0.03–0.48)	0.002		0.14 (0.04–0.54)	0.004	
Hispanic/Latino	2.01 (0.60–6.73)	0.258		1.85 (0.55–6.22)	0.32	
Mixed/Other	2.73 (0.68–10.98)	0.155		1.42 (0.32–6.42)	0.644	
**Age, y**						
18–34 (Millenials)	Reference		0.148	Reference		0.053
35–50 (Generation X)	1.83 (0.61–5.51)	0.280		1.22 (0.42–3.55)	0.710	
51+ (Baby Boomers)	2.67 (1.00–7.12)	0.050		2.96 (1.14–7.73)	0.027	
**Education**						
< High school	Reference		0.004	Reference		0.015
High School or GED	0.84 (0.15–4.73)	0.842		0.90 (0.15–5.38)	0.909	
Some College or 2 Year Degree	0.70 (0.13–3.73)	0.672		0.60 (0.11–3.40)	0.562	
Bachelor's Degree	0.18 (0.03–1.20)	0.076		0.22 (0.03–1.51)	0.122	
Any Post-Graduate Studies	0.05 (0.01–0.42)	0.007		0.11 (0.01–0.78)	0.02	
**Employment status**						
** **Not employed	Reference		0.147	Reference		0.285
** **Employed	0.60 (0.25–1.47)	0.264		0.86 (0.35–2.11)	0.740	
Student, not employed	0.17 (0.02–1.20)	0.076		0.21 (0.03–1.45)	0.113	
**Income (yearly), $**						
< 10,000	Reference		0.005	Reference		0.400
10,000–19,999	0.83 (0.26–2.66)	0.756		0.80 (0.25–2.51)	0.698	
20,000–39,999	1.14 (0.38–3.42)	0.821		0.85 (0.29–2.53)	0.773	
40,000–74,999	0.12 (0.01–1.18)	0.070		0.44 (0.07–2.64)	0.366	
≥ 75,000	0.14 (0.04–0.47)	0.001		0.34 (0.11–1.05)	0.062	
**Born outside the United States**	0.41 (0.10–1.60)	0.198	0.198	0.44 (0.12–1.67)	0.228	0.228
**Currently homeless**	3.06 (1.25–7.47)	0.014	0.014	2.07 (0.85–5.02)	0.108	0.108
**Relationship status**						
** **In a relationship	Reference			Reference		
Single	0.62 (0.24–1.64)	0.334	0.091	0.74 (0.29–1.92)	0.536	0.169
Other	4.40 (0.64–30.53)	0.133		3.85 (0.61–24.54)	0.153	
**ALCOHOL USE**						
**Interest in reducing amount of alcohol consumed**						
** **Not interested	Reference		0.003	Reference		0.022
Somewhat interested	2.73 (0.90–8.30)	0.077		2.08 (0.73–5.91)	0.168	
Moderately interested	7.21 (2.20–23.64)	0.001		3.50 (1.11–10.98)	0.032	
Extremely interested	7.48 (2.10–26.66)	0.002		6.29 (1.85–21.34)	0.003	
**Ever received treatment for alcohol use**	4.32 (0.79–10.38)	0.001	0.001	1.80 (0.78–4.18)	0.168	0.168
**OTHER SUBSTANCE USE**						
**Injected drugs, past 6 mo**	1.97 (0.70–5.49)	0.196	0.196	1.45 (0.53–3.94)	0.463	0.463
**Use of specific substances, past 6 mo**						
Methamphetamine	3.13 (1.30–7.52)	0.011	0.011	1.73 (0.74–4.03)	0.201	0.201
Powdered Cocaine	2.89 (1.15–7.29)	0.025	0.025	1.49 (0.59–3.72)	0.394	0.394
Crack Cocaine	4.22 (1.54–11.59)	0.005	0.005	1.67 (0.63–4.46)	0.304	0.304
Ecstasy	2.88 (1.10–7.57)	0.032	0.032	1.47 (0.54–4.03)	0.452	0.452
GHB	1.50 (0.46–4.96)	0.501	0.501	0.70 (0.23–2.13)	0.533	0.533
Ketamine	1.31 (0.27–6.47)	0.735	0.735	0.59 (0.15–2.36)	0.450	0.450
Poppers	1.65 (0.69–3.94)	0.261	0.261	0.68 (0.29–1.61)	0.378	0.378
Viagra	1.62 (0.60–4.36)	0.336	0.336	1.10 (0.42–2.94)	0.841	0.841
Marijuana	1.43 (0.60–3.41)	0.418	0.418	1.16 (0.49–2.72)	0.737	0.737
Cigarettes*	2.26 (0.89–5.73)	0.087	0.087	1.71 (0.69–4.19)	0.242	0.242
E-cigarettes*	2.52 (0.93–6.82)	0.070	0.070	0.87 (0.32–2.36)	0.790	0.790
**Ever received treatment for drug use**	3.05 (1.33–7.02)	0.009	0.009	2.06 (0.91–4.67)	0.083	0.083
**MENTAL AND PHYSICAL HEALTH**						
**Clinically Significant Depressive Symptoms**	3.28 (1.38–7.78)	0.007	0.007	1.54 (0.67–3.55)	0.310	0.310
**HIV status, self-report**						
Negative	Reference		0.621	Reference		0.634
Positive	1.34 (0.56–3.22)	0.504		1.03 (0.44–2.41)	0.946	
Unknown	0.49 (0.05–4.82)	0.539		0.34 (0.03–3.30)	0.350	
Undetectable viral load*	0.67 (0.15–2.93)	0.590	0.590	0.46 (0.11–1.97)	0.292	0.292
**Number of HIV tests, past 2 yr**						
Zero	Reference		0.294	Reference		0.887
One to three	2.29 (0.78–6.75)	0.133		1.30 (0.45–3.72)	0.626	
Four or more	1.34 (0.45–3.99)	0.598		1.14 (0.39–3.31)	0.810	
**Currently taking PrEP**	0.80 (0.22–2.92)	0.732	0.732	0.61 (0.17–2.21)	0.449	0.449
**HCV positive status, self-report**	2.59 (0.85–7.88)	0.094	0.094	4.19 (1.29–13.60)	0.017	0.017
**Ever had a sexually transmitted infection, past 6 mo**	3.31 (1.37–7.96)	0.008	0.008	2.76 (1.13–6.72)	0.025	0.025
**Type of sexually transmitted infection, past 6 mo**						
** **Syphilis	13.66 (3.48–53.54)	<0.001	<0.001	6.28 (1.64–24.10)	0.008	0.008
Gonnorhea	0.87 (0.26–2.89)	0.825	0.825	1.67 (0.57–4.92)	0.347	0.347
Chlamydia	1.29 (0.34–4.88)	0.709	0.709	4.21 (1.27–14.01)	0.019	0.019
Herpes (HSV)	1.06 (0.07–15.57)	0.967	0.967	1.10 (0.08–15.77)	0.943	0.943
Genital warts (HPV)	1.56 (0.28–8.70)	0.611	0.611	N/A†		
**SEXUAL BEHAVIORS**						
**Gender of sexual partners**						
Men	Reference			Reference		
Both Men and Women	1.99 (0.75–5.27)	0.167	0.167	1.20 (0.47–3.09)	0.703	0.703
**Number of sex partners, past 6 mo**						
Zero	Reference		0.011	Reference		0.022
One	2.88 (0.27–30.70)	0.390		4.71 (0.48–45.89)	0.181	
2–5	8.42 (1.14–62.43)	0.037		10.72 (1.44–79.59)	0.210	
6+	16.77 (2.23–126.24)	0.006		17.19 (2.29–129.07)	0.006	
**Number of male sex partners, past 6 mo**						
Zero	Reference		0.013	Reference		0.006
One	13.22 (1.83–95.57)	0.011		11.06 (1.53–79.78)	0.017	
2–5	15.08 (2.46–92.52)	0.004		21.85 (3.55–134.38)	0.001	
6+	20.93 (3.44–127.46)	0.001		21.64 (3.56–131.42)	0.001	
**Any condomless anal intercourse, past 6 mo**	0.66 (0.27–1.59)	0.349	0.349	0.58 (0.24–1.41)	0.229	0.229
**Any insertive condomless anal intercourse, past 6 mo**	0.75 (0.32–1.78)	0.519	0.519	0.53 (0.23–1.25)	0.147	0.147
**Any receptive condomless anal intercourse, past 6 mo**	0.65 (0.28–1.49)	0.305	0.305	0.67 (0.29–1.53)	0.337	0.337
**Any serodiscordant condomless anal intercourse, past 6 mo**	1.72 (0.73–4.07)	0.218	0.218	1.23 (0.53–2.86)	0.626	0.626

*Use of cigarettes and e-cigarettes only asked for a subset of participants (n = 208); undetectable viral load only available for HIV-positive participants who reported their most recent viral load (n = 66).

^†^Odds ratio not calculable due to no outcome variability in all strata of participant characteristic.

### Multivariable analyses for hazardous alcohol consumption

In RDS-adjusted multivariable analyses, participants who identified as Hispanic/Latino (adjusted odds ratio [AOR] = 6.27; 95% CI = 1.29–30.63), and mixed or other race (AOR = 15.22; 95% CI = 1.31–121.58) had significantly greater odds of hazardous drinking compared to white MSM (Table 5). In addition, those who reported being moderately (AOR = 11.57; 95% CI = 2.62–51.05) or extremely interested (AOR = 14.68; 95% CI = 1.66–130.03) in reducing their alcohol consumption, ever receiving treatment for alcohol use (AOR = 6.97; 95% CI = 1.61–30.26), using ecstasy (AOR = 8.13; 95% CI = 1.67–39.47), reporting a recent diagnosis of syphilis (AOR = 142.14; 95% CI = 20.15–1002.57), and having more than 5 male sexual partners (AOR = 10.27; 95% CI = 1.02–103.47) had greater odds of hazardous alcohol consumption. Lower odds of hazardous alcohol consumption was significantly associated with higher educational attainment (bachelor's degree AOR = 0.12; 95% CI = 0.02–0.98; any post-graduate studies AOR = 0.01; 95% CI = 0.00–0.22, compared to those who did not complete high school) and not being in a relationship (AOR = 0.29; 95% CI = 0.09–0.95).

**Table 5:** RDS-weighted multivariable associations with hazardous alcohol consumption among alcohol using men who have sex with men: San Francisco, CA; March 2015—June 2017 (n = 239).

Characteristic	Hazardous Alcohol Consumption		
	OR (95% CI)	*p*-value	Wald *p*-value
DEMOGRAPHIC CHARACTERISTICS			
**Race/Ethnicity**			
White	Reference		0.037
Black/African American	2.68 (0.76–9.48)	0.125	
Asian/Pacific Islander	0.74 (0.09–5.89)	0.776	
Hispanic/Latino	6.27 (1.29–30.63)	0.023	
Mixed/Other	15.22 (1.31–121.58)	0.010	
**Age, y**			
18–34 (Millenials)	Reference		0.974
35–50 (Generation X)	0.91 (0.18–4.74)	0.913	
51+ (Baby Boomers)	0.84 (0.18–3.92)	0.822	
**Education**			
< High school	Reference		0.058
High School or GED	0.27 (0.04–1.81)	0.177	
Some College or 2 Year Degree	0.45 (0.10–1.94)	0.281	
Bachelor's Degree	0.12 (0.02–0.98)	0.048	
Any Post-Graduate Studies	0.01 (0.00–0.22)	0.005	
**Clinically Significant Depressive Symptoms**	1.16 (0.39–3.42)	0.793	0.793
**Relationship status**			
In a relationship	Reference		0.113
Single	0.29 (0.09–0.95)	0.041	
Other	0.88 (0.02–43.89)	0.949	
**Interest in reducing amount of alcohol consumed**			
Not interested	Reference		0.007
Somewhat interested	3.27 (0.79–13.54)	0.102	
Moderately interested	11.57 (2.62–51.05)	0.001	
Extremely interested	14.68 (1.66–130.03)	0.016	
**Ever received treatment for alcohol use**	6.97 (1.61–30.26)	0.010	0.010
**Used crack cocaine, past 6 mo**	2.29 (0.67–7.87)	0.185	0.185
**Used ecstasy, past 6 mo**	8.13 (1.67–39.47)	0.010	0.010
**Contracted Syphilis, past 6 mo**	142.14 (20.15–1002.57)	<0.001	<0.001
**Number of male sex partners, past 6 mo**			
Zero	Reference		0.156
One	4.29 (0.24–76.9)	0.321	
2–5	6.08 (0.69–53.43)	0.103	
6+	10.27 (1.02–103.47)	0.048	

In the interaction models, there were significant interaction effects between race/ethnicity and interest in reducing alcohol use, past receipt of treatment for alcohol use, use of ecstasy, and reporting a recent diagnosis of syphilis (S1 Table). Specifically, there were strong associations between having high interest in reducing the amount of alcohol consumed among white (moderately interested AOR = 370.21; 95%CI = 26.22–5226.39; extremely interested AOR = 531.90; 95%CI = 30.63–9236.86) and Asian/Pacific Islander participants (extremely interested AOR = 119.63; 95%CI = 3.81–3758.25), but not among Black/African American or Hispanic/Latino participants. In addition, past receipt of treatment was associated with hazardous alcohol consumption among Black/African American participants (AOR = 18.74; 95%CI = 2.43–144.46) but not among other racial/ethnic groups and ecstasy use was associated with hazardous alcohol consumption among both Black/African American (AOR = 49.29; 95%CI = 1.74–1397.43) and Hispanic/Latino participants (AOR = 84.71; 95%CI = 8.07–889.65) but not among other groups. Lastly, reporting a recent diagnosis of syphilis was associated with hazardous alcohol consumption among white participants (AOR = 171.42; 95%CI = 1680.28) but not among Hispanic/Latino participants.

### Multivariable analyses for binge drinking

As shown in Table 6, in RDS-adjusted multivariable analyses, reporting a recent chlamydia diagnosis (AOR = 4.19; 95% CI = 1.04–16.90), having interest in reducing the amount of alcohol consumed (somewhat interested AOR = 3.54; 95% CI = 1.15–10.90; moderately interested AOR = 4.76; 95% CI = 1.34–16.91; extremely interested AOR = 10.01; 95% CI = 2.24–44.68), and greater number of male sex partners (having 2–5 male partners AOR = 29.78; 95% CI = 2.83–313.90; having more than 5 male partners AOR = 24.05; 95% CI = 2.05–282.18) were associated with at least weekly binge drinking. In addition, lower odds of at least weekly binge drinking were associated with having any post-graduate studies (AOR = 0.06; 95% CI = 0.01–0.56).

**Table 6:** RDS-weighted multivariable associations with weekly or more frequent binge drinking among alcohol using men who have sex with men: San Francisco, CA; March 2015—June 2017 (n = 243).

Characteristic	Weekly or More Frequent Binge Drinking		
	OR (95% CI)	*p*-value	Wald *p*-value
DEMOGRAPHIC CHARACTERISTICS			
**Race/Ethnicity**			
White	Reference		0.464
Black/African American	0.83 (0.30–2.30)	0.721	
Asian/Pacific Islander	0.36 (0.08–1.63)	0.182	
Hispanic/Latino	1.82 (0.34–9.78)	0.485	
Mixed/Other	1.81 (0.32–10.35)	0.503	
**Age, y**			
18–34 (Millenials)	Reference		0.857
35–50 (Generation X)	0.75 (0.22–2.56)	0.643	
51+ (Baby Boomers)	0.99 (0.32–3.09)	0.985	
**Education**			
< High school	Reference		0.083
High School or GED	0.73 (0.14–3.68)	0.699	
Some College or 2 Year Degree	0.38 (0.08–1.75)	0.215	
Bachelor's Degree	0.21 (0.03–1.31)	0.094	
Any Post-Graduate Studies	0.06 (0.01–0.56)	0.014	
**Clinically Significant Depressive Symptoms**	0.84 (0.34–2.05)	0.703	0.703
**Interest in reducing amount of alcohol consumed**			
Not interested	Reference		0.010
Somewhat interested	3.54 (1.15–10.90)	0.028	
Moderately interested	4.76 (1.34–16.91)	0.016	
Extremely interested	10.01 (2.24–44.68)	0.003	
**HCV positive status, self-report**	2.54 (0.52–12.38)	0.246	0.246
**Contracted Syphilis, past 6 mo**	5.06 (0.83–30.67)	0.078	0.078
**Contracted Chlamydia, past 6 mo**	4.19 (1.04–16.90)	0.044	0.044
**Number of male sex partners, past 6 mo**			
Zero	Reference		0.017
One	7.67 (0.50–117.58)	0.143	
2–5	29.78 (2.83–313.90)	0.005	
6+	24.05 (2.05–282.18)	0.012	

In the interaction models, there were significant interaction effects between race/ethnicity and number of recent male sex partners (S2 Table). Specifically, the relationship between number of male sex partners and odds of reporting weekly or more binge drinking was positive among Black/African American (2–5 partners AOR = 45.55; 95%CI = 5.11–406.13; 6+ partners AOR = 53.55; 95%CI = 5.20–551.13) and mixed or other race participants (2–5 partners AOR = 118.91; 95%CI = 2.16–6547.14; 6+ partners AOR = 229.31; 95%CI = 4.32–12174.01), negative among Hispanic/Latino participants (2–5 partners AOR = 0.01; 95%CI = 0.00–0.41; 6+ partners AOR = 0.01; 95%CI = 0.00–0.44), and null among white and Asian/Pacific Islander participants.

## Discussion

This study aimed to evaluate the prevalence of heavy alcohol use patterns, and explore correlates of heavy alcohol consumption in a diverse sample of alcohol-using MSM recruited by RDS. We observed a high prevalence of hazardous alcohol consumption and binge drinking. These results are broadly consistent with the high rates of binge drinking observed in National HIV Behavioral Surveillance (NHBS) data (NHBS did not measure AUDIT scores), which observed that binge drinking at least weekly was reported by 43% of MSM in San Francisco [8]. Taken together, these consistent findings highlight how heavy alcohol use remains a major public health issue among MSM.

Of note, our study also observed that while the majority of alcohol-using MSM (65%) had tried to stop or reduce their alcohol consumption in their lifetime, only one-third had ever utilized alcohol use disorder treatment. Moreover, a high percentage of alcohol-using MSM had reported having a goal to reduce alcohol in a controlled manner, and being interested in reducing their alcohol consumption, which was in turn, independently associated with both hazardous consumption and at least weekly binge-drinking. The ubiquity of heavy drinking patterns among alcohol-using MSM, and the level of interest in reducing alcohol consumption suggest that there’s a great need to expand current alcohol intervention strategies among MSM, particularly heavy drinking MSM, beyond traditional substance use disorder treatment settings. One strategy that remains underutilized and underexplored is the use and development of pharmacotherapy for MSM. We observed that only 6% of alcohol-using MSM with hazardous alcohol consumption had used pharmacotherapy, which is lower than uptake of medically assisted alcohol treatment among individuals with alcohol use disorders in the United States, estimated at just below 10% [4,5]. It is unclear why use of pharmacotherapy among hazardous MSM drinkers is less common. We speculate that the high proportion of MSM who prioritized goals related to reducing alcohol use over abstinence may play a role. Indeed, MSM may be less likely to completely abstain from alcohol than heterosexual men [44], emphasizing the need to consider alternative treatment outcomes including reductions in drinking. Future pharmacotherapy intervention trials should consider alternate treatment goals consistent with their target study populations. To date, limited pharmacotherapy trials have explored this harm-reduction approach among MSM with treatment goals pertaining to reduction of use. A double-blind, placebo-controlled pilot study among dual methamphetamine and binge-drinking MSM showed that the use of oral naltrexone on an as-needed, intermittent basis is significantly associated with reductions in binge drinking days among participants who took their medications at least thrice weekly [45]. An efficacy trial, entitled “The Say When Study” is currently underway to evaluate intermittent naltrexone’s efficacy to address binge drinking and alcohol-associated sexual risk behaviors among binge-drinking MSM [37]. Ultimately, efforts to develop different types of evidence-based interventions, including pharmacotherapy, with reduction end-points may be needed for alcohol-using MSM in order to effectively reduce the morbidity and mortality associated with heavy alcohol use.

Notably, we also observed significant racial/ethnic and sociodemographic health disparities with heavy alcohol use. Hispanic/Latino MSM, as well as other race/mixed race MSM had greater odds of hazardous alcohol use, while lower educational attainment was associated with greater odds of both hazardous alcohol use and binge drinking. Moreover, we observed significant interaction effects between race/ethnicity and interest in reducing alcohol, past receipt of alcohol treatment, use of ecstasy, syphilis diagnosis, and number of male partners. These findings highlight the differential effects of behavioral and clinical characteristics in different racial/ethnic groups with respect to heavy alcohol use. These findings are broadly consistent with other analyses for the general adult population. In light of these disparities, efforts for targeted screening may be needed to help alleviate the disproportionate burden of heavy alcohol use observed in these subpopulations. Moreover, developing culturally-tailored interventions specific to the needs of different racial/ethnic groups may be needed, given the fact that the characteristics associated with hazardous alcohol use and binge drinking was moderated by race/ethnicity.

Additionally, we found significant associations between increased hazardous alcohol consumption, binge drinking, and HIV risk, including recent STIs and multiple sexual partners. Our finding corroborate linkages observed between heavy alcohol use patterns and HIV-related risk factors among MSM [10,11,18,35,46–59]. As MSM comprise the majority of new HIV infections in the United States, and evidence supports the important role heavy alcohol use plays in HIV-related risk behaviors, research jointly addressing alcohol use and HIV risk among MSM should be expanded. For example, one study among substance-using MSM found that a brief behavioral intervention involving personalized cognitive counseling may help reduce sexual risk among a sub-group of MSM who are not dependent on alcohol and other substances, while also having collateral benefits in reducing alcohol use [60]. Additional efforts to develop and evaluate interventions that aim to address the overlap between alcohol use and HIV risk interventions for MSM are therefore immensely needed.

We also observed unexpected correlates of heavy alcohol use. For example, MSM in relationships had greater odds of hazardous drinking. Unfortunately, we did not collect information on the duration of relationships and the cross-sectional design does not provide the temporal sequence between hazardous drinking and relationship status. It is plausible that some heavy alcohol using participants have developed relationships with partners who are also heavy drinkers. Concordance in drinking patterns in relationships have been associated with greater time spent drinking with partners and greater levels of happiness in some samples [61]. Qualitative and other empirical explorations are needed on the drinking patterns of partners of alcohol-using MSM to establish whether concordance is highly prevalent and how drinking concordance impacts hazardous alcohol use.

This study has several limitations. The prevalence of white participants and college-educated participants was lower than broader MSM samples in San Francisco [8]. It is plausible that the study eligibility criteria for recent alcohol use may have led to recruitment of a population that is demographically different from broader MSM samples. Indeed, in NHBS, the prevalence of binge drinking was lower among white MSM (44% versus 52% for African American and 58% for Latino MSM) and college-educated MSM (50% versus 54% for MSM with a high school diploma or less)[8]. Furthermore, our study may have included individuals who are more marginalized, have fewer resources, and/or have more time to participate in the study. Prior RDS studies noted greater recruitment of participants with lower education or income [62,63] compared to non-RDS samples. Indeed, researchers have observed challenges in using RDS to recruit MSM with higher socioeconomic status (SES) in San Francisco and elsewhere [23,25]. Empirical comparisons between RDS, time location sampling, and snowball sampling studies for MSM have noted that RDS is able to recruit greater number of participants with lower SES than these other study designs [64]. Another RDS study among black MSM in San Francisco also resulted in a sample that was primarily comprised of participants (RDS-adjusted prevalence: 61%) with annual income less than $10,000 [32]. Comparison between traditional RDS and “Web-based RDS”, whereby all study procedures—including behavioral assessment and recruitment—occurred on the Web, observed that Web-based RDS generally recruited participants from higher SES backgrounds [62]. Future studies exploring and comparing the utility of Web-based RDS may be worthwhile among alcohol-using MSM, to establish whether one method is more effective than the other for recruiting more representative samples of this population.

Another limitation is self-reported data, which are prone to social desirability and recall bias; however, we employed ACASI in an effort to mitigate these biases [34]. Additionally, our findings may not be generalizable to other MSM populations who do not use alcohol and to those who live outside of the San Francisco Bay Area. Moreover, we observed wide confidence intervals for some point estimates in our multivariable analyses due to the sample size of our study (e.g., reporting recent syphilis diagnoses and odds of hazardous alcohol consumption). Those findings should be interpreted with caution. Studies with larger sample sizes may be needed to confirm and estimate with greater precision the relationships between our outcomes and the correlates we identified in our study.

Despite limitations, this study expands our understanding of alcohol use patterns and correlates of problematic alcohol use in a population that has been noted to have high morbidity associated with alcohol use. Moreover, this study corroborates the known linkages between HIV-related sexual risk behaviors, highlighting the immense need to develop more interventions for MSM that jointly address heavy alcohol use and HIV/STI risk, as this population may doubly benefit from such interventions. Finally, our study documented significant racial/ethnic and sociodemographic health disparities and significant interaction effects between race/ethnicity and different risk factors, emphasizing the importance of expanding culturally-tailored and targeted screening and intervention strategies to address heavy alcohol use across disproportionately impacted communities.

## Supporting information

S1 Table RDS-weighted multivariable associations with hazardous alcohol consumption by race/ethnicity among alcohol using men who have sex with men: San Francisco, CA; March 2015—June 2017. (DOCX)

S2 Table RDS-weighted multivariable associations with weekly or more frequent binge drinking by race/ethnicity among alcohol using men who have sex with men: San Francisco, CA; March 2015—June 2017. (DOCX)

S1 File Anonymized study dataset. (CSV)
