# Supplementary material for: Prevalence and correlates of hazardous alcohol consumption and binge drinking among men who have sex with men (MSM) in San Francisco
Source: PLoS One. 2018 Aug 17;13(8):e0202170. doi: 10.1371/journal.pone.0202170 (PMC6097698; doi:10.1371/journal.pone.0202170)
Supplement: S1 Table — (DOCX) [file pone.0202170.s001.docx]

**S1 Table: RDS-Weighted Multivariable Associations with Hazardous Alcohol Consumption by Race/Ethnicity Among Alcohol Using Men Who Have Sex with Men: San Francisco, CA; March 2015 - June 2017†**

|  | **White** | |  | **Black/African American** | |  | **Asian/Pacific Islander** | |  | **Hispanic/Latino** | |  | **Mixed/Other** | |  |
| --- | --- | --- | --- | --- | --- | --- | --- | --- | --- | --- | --- | --- | --- | --- | --- |
| **Characteristic** | **OR** | **(95% CI)** |  | **OR** | **(95% CI)** |  | **OR** | **(95% CI)** |  | **OR** | **(95% CI)** |  | **OR** | **(95% CI)** | **Interaction Effect** |
| **DEMOGRAPHIC CHARACTERISTICS** |  |  |  |  |  |  |  |  |  |  |  |  |  |  |  |
| **Interest in reducing amount of alcohol consumed^‡^** |  |  |  |  |  |  |  |  |  |  |  |  |  |  |  |
| Not or somewhat interested | Reference | |  | Reference | |  | Reference | |  | Reference | |  | Reference | | 0.001 |
| Moderately interested | 370.21 | (26.22-5226.39)* |  | 1.45 | (0.29-7.24) |  | 3.46 | (0.12-98.82) |  | 8.06 | (0.24-276.25) |  | N/A^§^ | |  |
| Extremely interested | 531.90 | (30.63-9236.86)* |  | 0.51 | (0.07-3.84) |  | 119.63 | (3.81-3758.25)* | | 13.32 | (0.04-4149.67) |  | N/A^§^ | |  |
| **Ever received treatment for alcohol use** | 3.41 | (0.52-22.36) |  | 18.74 | (2.43-144.46)* |  | 36.56 | (0.98-1369.01) |  | 0.81 | (0.06-11.12) |  | 3.41 | (0.52-22.36) | 0.163 |
| **Used ecstasy, past 6 mo** | 2.78 | (0.19-41.22) |  | 49.29 | (1.74-1397.43)* | | 1.57 | (0.08-30.61) |  | 84.71 | (8.07-889.65)* |  | 0.25 | (0.01-12.30) | 0.080 |
| **Contracted Syphilis, past 6 mo** | 171.42 | (17.49-1680.28)* |  | N/A^§^ | |  | N/A^§^ | |  | 2.48 | (0.22-28.52) |  | N/A^§^ | | 0.080 |

*p<0.05

†Each demographic characteristic assessed in separate model: n=232 for Interest in reducing amount of alcohol consumed; n=234 for Ever received treatment for alcohol use; n=239 for Used ecstasy, past 6 mo; n=235 for Contracted Syphilis, past 6 mo.

‡Due to data sparsity and to allow for consistent interpretation across racial/ethnic subgroups, "Not interested" and "Somewhat interested" were collapsed into a single category and used as the reference level. These two categories were not significantly different at p<0.05 in the final model presented in Table 5.

§No outcome variability among covariate values, so no estimates available.
