# Supplementary material for: Prevalence and correlates of hazardous alcohol consumption and binge drinking among men who have sex with men (MSM) in San Francisco
Source: PLoS One. 2018 Aug 17;13(8):e0202170. doi: 10.1371/journal.pone.0202170 (PMC6097698; doi:10.1371/journal.pone.0202170)
Supplement: S2 Table — (DOCX) [file pone.0202170.s002.docx]

**S2 Table: RDS-Weighted Multivariable Associations with Weekly or More Frequent Binge Drinking by Race/Ethnicity Among Alcohol Using Men Who Have Sex with Men: San Francisco, CA; March 2015 - June 2017**

|  | **White** | |  | **Black/African American** | |  | **Asian/Pacific Islander** | |  | **Hispanic/Latino** | |  | **Mixed/Other** | |  |
| --- | --- | --- | --- | --- | --- | --- | --- | --- | --- | --- | --- | --- | --- | --- | --- |
| **Characteristic** | **OR** | **(95% CI)** |  | **OR** | **(95% CI)** |  | **OR** | **(95% CI)** |  | **OR** | **(95% CI)** |  | **OR** | **(95% CI)** | **Interaction Effect** |
| **DEMOGRAPHIC CHARACTERISTICS** |  |  |  |  |  |  |  |  |  |  |  |  |  |  |  |
| **Number of male sex partners, past 6 mo^†^** |  |  |  |  |  |  |  |  |  |  |  |  |  |  |  |
| 0-1 | Reference | |  | Reference | |  | Reference | |  | Reference | |  | Reference | | 0.003 |
| 2-5 | 4.92 | (0.90-26.99) |  | 45.55 | (5.11-406.13)* |  | 0.69 | (0.07-6.74) |  | 0.01 | (0.00-0.41)* |  | 118.91 | (2.16-6547.14)* |  |
| 6+ | 3.58 | (0.50-25.40) |  | 53.53 | (5.20-551.13)* |  | N/A^‡^ | |  | 0.01 | (0.00-0.44)* |  | 229.31 | (4.32-12174.01)* |  |

*p<0.05

†Due to data sparsity and to allow for consistent interpretation across racial/ethnic subgroups, zero male sex partners and one male sex partner were collapsed into a single category and used as the reference level. These two categories were not significantly different at p<0.05 in the final model presented in Table 6.

‡No outcome variability among covariate values, so no estimates available.
